# Association Between Renalase Gene Polymorphism (rs2296545) and Hypertension in Egyptian Chronic Kidney Disease Patients

**DOI:** 10.7759/cureus.47903

**Published:** 2023-10-29

**Authors:** Mohamed H Khater, Dalia M Abd EL-Hassib, Jehan H Sabry, Rania M Elkilany, Seham G Ameen

**Affiliations:** 1 General Surgery, Nile Health Insurance Hospital, Shubra El-Kheima, EGY; 2 General Surgery, Northwick Park Hospital, Harrow, GBR; 3 Clinical and Chemical Pathology, Faculty of Medicine Benha University, Benha, EGY

**Keywords:** egypt, cc genotype, c allele renalase, pcr-rflp, hypertensive ckd, end-stage renal disease (esrd), chronic kidney disease (ckd)

## Abstract

Background

Renalase gene polymorphisms are associated with an increased risk of essential hypertension, chronic kidney disease (CKD), heart disease, diabetes, and stroke. One of these polymorphisms is a common missense (rs2296545) polymorphism, which was reported to be related to hypertension. The aim of this work was to investigate the possible relation between renalase gene polymorphism (rs2296545) and hypertension in patients with CKD patients.

Subjects and methods

Ninety patients were included in this case-control study: 30 normotensive CKD patients, 30 hypertensive CKD patients, and 30 apparently healthy controls. Genomic deoxyribonucleic acid (DNA) was extracted from peripheral whole blood, and renalase gene (rs2296545) polymorphism was genotyped in all patients and controls by polymerase chain reaction-restriction fragment length polymorphism (PCR-RFLP). Odds ratios (OR) and their 95% CIs were calculated.

Results

We found that the CC genotype and the C allele renalase (rs2296545) were statistically associated with the risk of CKD (OR= 9.4; 95%CI 1.2-7.2; P= 0.036) and (OR= 3.78; 95%CI 1.57-9.08; P= 0.003), respectively. There was a statistically significant difference between the hypertensive CKD patients and the controls regarding the CC genotypes and the C allele, (26.7% versus 3.3%, P= 0.018) and (40% versus 11.7%, P< 0.001) for the CC genotype and the C allele, respectively. The mean values of systolic and diastolic blood pressure were higher in the normotensive CKD patients with the CC genotype compared to other genotypes (P= 0.014 and P= 0.022, respectively) and also were higher in hypertensive CKD patients with the CC genotype when compared to other genotypes (P= 0.001 for both).

Conclusion

This study demonstrated a statistically significant increase in the renalase gene (rs2296545) CC genotype and the C allele in CKD patients, especially hypertensive CKD.

## Introduction

Chronic kidney disease (CKD) is a global disorder with many etiologies. Genetic etiology is an important predictor of CKD, especially for those who end up with end-stage renal disease (ESRD) [1]. Also, hypertension (HTN) is another important and leading cause of CKD [2,3]. Both CKD and HTN share the same risk factors, and they affect each other, and sometimes, it becomes difficult to decide which is the cause and its effect [2,4].

Renalaseis is a 342-amino-acid flavoprotein enzyme that regulates cardiac function and blood pressure [5,6]. While circulating in the blood and regulating blood pressure by increasing catecholamines degradation [5,6], its deficiency may be linked to cardiovascular and stroke diseases in addition to CKD [7,8].

One of the renalase polymorphisms is a common missense rs2296545, in that there is a substitution from glutamic acid to aspartic acid (Glu 37 Asp) at codon 37 [9]. This affects renalase enzyme function. Much research has proved the association between the C allele and the risk for the development of HTN, especially in Caucasians [7]. rs2296545 CC is linked with a series of cardiac pathologies, such as cardiac hypertrophy and ischemia [10]. Also, it is observed that renalase enzyme was upregulated in hemodialysis patients, especially those with a long time history of HTN [11].

This study aimed to assess the genetic association of renalase gene (rs2296545) polymorphism and HTN among Egyptian CKD patients.

## Materials and methods

Setting and population

A total of 90 participants were included in this case-control study; of these, 60 were CKD patients and 30 were age- and gender-matched healthy control participants. The included CKD patients were further divided into two groups: the first included 30 hypertensive patients, and the other included 30 normotensive patients. The included patients were recruited from the Nephrology Department and Renal Dialysis Unit of the Internal Medicine Department at Benha University Hospital, Benha, Egypt, between March 2017 and May 2018. All participants provided written informed consent before participating in this study. The Research Ethics Committee of Benha University Faculty of Medicine approved the protocol of the study (approval number: MS.5.3.2017). A full history and clinical examination were applied to all participants.

CKD diagnosis was based on the Kidney Disease Outcomes Quality Initiative (KlDOQI) clinical practice guidelines for CKD, which provided evidence-based guidelines for all stages of CKD [12]. Glomerular filtration rate (GFR) was estimated using the Cockcroft-Gault (CG) formula [13], which estimates GFR in ml/minute. The HTN was diagnosed when a person’s systolic blood pressure (SBP) in the office or clinic was ≥140 mm Hg and/or their diastolic blood pressure (DBP) was ≥90 mm Hg following repeated examination, according to American Heart Association guidelines [14].

We included patients of both sexes who fulfill the following criteria: (i) Aged between 40 and 60 years; (ii) CKD patients with estimated GFR (eGFR) < 60 ml/min/1.73 m^2^ for three months or more; (iii) Non-diabetic; (iv) No evidence of hereditary kidney disease as solitary kidney; (v) Free from immune complex disease; (vi) Able to understand the information about all study procedures and to give free written approval consent prior enrollment in the study, and (vii) BP > 140/90 mmHg or patient on antihypertensive drugs in hypertensive CKD group and BP < 140/90 mmHg without treatment in normotensive CKD group.

Molecular assessment

DNA Extraction

Genomic DNA was extracted from peripheral whole blood on EDTA by spin column method in concordance with blood genome DNA extraction kits protocol (Gene JET Whole Blood Genomic DNA Purification Mini Kit; ThermoFisher Scientific Inc., Waltham, Massachusetts, United States) [15]. The extracted DNA was frozen at -80°C until use.

Detection of Renalase Gene Polymorphism (rs2296545)

Using polymerase chain reaction (PCR)-restriction fragment length polymorphism (RFLP), renalase gene single nucleotide polymorphism (SNP) genotypes (rs2296545) were detected [16]. Then, amplification of the extracted DNA was obtained; the forward primer was GGAAGTCCCCGATCACGTGAC, and the reverse primer was TGCTGTGTGGGACAAGGCTGA.

PCR was done according to the manufacturer structure (Applied Biosystem Veriti™ 96-Well PCR Thermal Cycling Block S/N 2990226734; ThermoFisher Scientific Inc.). Initial denaturation was at 95°C for five minutes for one cycle, denaturation at 94°C for 30 seconds, annealing at 58°C for 30 seconds, extension at 72°C for one minute for 35 cycles, and final extension at 72°C for five minutes.

PCR products were added to the loading dye and then separated by gel electrophoresis 2% agarose gel and ethidium bromide was used for band staining. Then, it was visualized by ultraviolet light (Biometra Transilluminator; Analytik Jena AG, Jena, Germany). The amplified products were analyzed with restriction enzyme (Eco 81I) that is unique for the target polymorphism renalase (rs2296545)with incubation for 16 hours with (Eco 81I).

Statistical analysis

Our data were analyzed by IBM SPSS Statistics for Windows, Version 20.0 (Released 2011; IBM Corp., Armonk, New York, United States). Shapiro-Wilks test was used to test normality. Mean ± standard deviation and range were used to describe the normally distributed quantitative data, and median and interquartile range were used to describe the not-normally distributed quantitative data, while numbers and percentages were used to show the categorical data. OR and 95%CIs were calculated. ANOVA test was used to statistically analyze the mean difference between the groups and the chi-square test was applied to compare qualitative categorical variables between groups. P <0.05 was considered significant.

## Results

**Figure 1:**
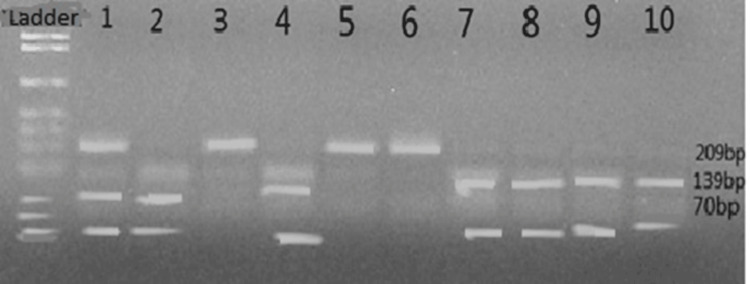
Renalase gene analysis (rs2296545) polymorphism Lane 1 is (CG) heterozygous (70, 139, and 209 bp); Lane 3, 5, and 6 (GG) homozygous (209 bp); Lane 2,4, 7, 8, 9, and 10 (CC) homozygous (70 and 139 bp).

In the present study, after digestion of rs2296545 with corresponding restriction endonuclease (Eco 81I), three genotypes were found: CC (139 and 70 bp), GG (209 bp), and CG (209,139 and 70 bp) (Figure 1).

The genotypes and allele frequency of rs2296545 CKD patients and controls are shown in Table 1. After comparisons between the two groups, the frequencies of the CC genotype and C allele showed a statistically significant increase in the CKD group in comparison to the control group (P=0.036 and P= 0.003 for the CC genotype and C allele, respectively). However, there is no statistically significant difference in CG genotype frequency between the studied groups (P=0.22). Subjects with renalase CC genotype and carriers of the C allele were significantly more likely to have CKD (OR=9.4, 95%CI= 1.2-77.2) and (OR=3.78, 95%CI= 1.57- 9.08), respectively. C allele represents a separate risk factor independent of CKD.

**Table 1:** Comparison between CKD patients group and control group regarding genotypes and allelic frequency of renalase (rs2296545) CKD: chronic kidney disease; OR: odds ratio; Ref: reference.

Variable		CKD patients (n=60)		Controls (n=30)		OR (95%CI)	P-value
		No.	%	No.	%		
rs 2296545 genotypes	GG	33	55.0	24	80.0	Ref	----
	CG	14	23.3	5	16.7	2.03 (0.65-6.4)	0.22
	CC	13	21.7	1	3.3	9.4 (1.2-77.2)	0.036
Allele	G	80	66.7	53	88.3	Ref	0.003
	C	40	33.3	7	11.7	3.78 (1.57-9.08)	

The genotypes and allele frequency of rs2296545, normotensive CKD patients, hypertensive CKD patients, and controls are shown in Table 2. The frequency of CG genotype(P=0.54) and CC genotype (P=0.105) between the control group and the normotensive CKD group did not differ in a statistically meaningful way. However, there was a statistically significant increase in the frequency of the C allele in the normotensive CKD group compared to the control group (P=0.041).

**Table 2:** Comparison between the studied groups regarding genotypes and allelic frequency of renalase (rs2296545) CKD: chronic kidney disease; No: number; OR: odds ratio; Ref: reference.

Variable		Controls (n=30)		Normotensive CKD patients (n=30)		OR (95%CI)	P	Hypertensive CKD patients (n=30)		OR (95%CI)	P-value
		No.	%	No.	%			No.	%		
rs 2296545 genotypes	GG	24	80.0	19	63.3	Ref	----	14	46.7	Ref	----
	CG	5	16.7	6	20.0	1.51 (0.4-5.7)	0.54	8	26.7	2.7 (0.75-10)	0.12
	CC	1	3.3	5	16.7	6.3 (0.67-58.7)	0.105	8	26.7	13.7 (1.5-121.4)	0.018
Allelic frequency	G	53	88.3	44	73.3	Ref	0.041	36	60.0	Ref	<0.001
	C	7	11.7	16	26.7	2.75 (1.04-7.3)		24	40.0	5.04 (1.96-12.9)	

The frequency of the CC genotype showed a statistically meaningful significant increase in the hypertensive CKD group compared to the control group (P=0.018). The frequency of the C allele showed a statistically highly significant increase in the hypertensive CKD group compared to the control group (P<0.001).

Renalase CC genotype and C allele carriers had a considerably higher risk of developing hypertensive CKD (OR=13.7, 95%CI 1.5- 121.3) and (OR=5.04, 95%CI 1.96- 12.9), respectively. CG genotype frequency between the hypertensive CKD group and the control group did not differ in a statistically significant way from the control group (P=0.12).

The mean SBP and DBP showed a statistically significant increase in normotensive CKD patients with renalase CC genotype compared to other renalase genotypes (P= 0.014 and 0.002. respectively) (Table 3).Additionally, the same results were obtained in the hypertensive CKD patients(Table 4).

**Table 3:** Comparison between renalase (rs2296545) genotypes in blood pressure in the studied normotensive CKD patients' group ANOVA: analysis of variance; SBP: systolic blood pressure; DBP: diastolic blood pressure; CKD: chronic kidney disease.

Group		n.	SBP<140mmHg				ANOVA	P-value
			Mean	± SD	Min.	Max.		
Genotype	GG	33	122.4	9.69	100.0	140.0	4.61	0.014
	CG	14	126.4	10.81	100.0	140.0		
	CC	13	133.1	13.15	100.0	150.0		
			DBP<90mmHg					
Genotype	GG	33	74.2	5.01	70.0	80.0	4.1	0.022
	CG	14	75.7	7.55	70.0	90.0		
	CC	13	80.0	7.07	70.0	90.0		

**Table 4:** Comparison of renalase (rs2296545) genotypes in blood pressure in hypertensive CKD patients ANOVA: analysis of variance; SBP: systolic blood pressure; DBP: diastolic blood pressure; CKD: chronic kidney disease.

Group		n.	SBP (mmHg)				ANOVA	P-value
			Mean	± SD	Min.	Max.		
Genotype	GG	14	124.2	6.46	120.0	140.0	9.63	0.001
	CG	8	131.2	6.40	120.0	140.0		
	CC	8	138.7	9.91	130.0	150.0		
			DBP (mmHg)					
Genotype	GG	14	72.8	4.68	70.0	80.0	8.79	0.001
	CG	8	78.7	8.34	70.0	90.0		
	CC	8	83.7	5.17	80.0	90.0		

## Discussion

The study focused on analyzing the rs2296545 polymorphism within the renalase gene, aiming to determine its potential association with CKD and HTN. After enzymatic digestion, three genotypes, CC, GG, and CG, were identified. Comparisons between CKD patients and controls revealed a significant increase in the frequency of the CC genotype and the C allele in the CKD group. The C allele was found to be an independent risk factor for CKD development.

Further analysis of the genotypes and allele frequencies in normotensive and hypertensive CKD patients compared to controls showed that the frequency of the CC genotype and the C allele was significantly higher in hypertensive CKD patients. These carriers were at a notably higher risk of developing hypertensive CKD. The findings highlight the potential role of the rs2296545 polymorphism in the renalase gene as a risk factor for CKD, particularly in the presence of HTN. These results suggest that this genetic variation may be a potential marker for assessing susceptibility to CKD, emphasizing the importance of genetic factors in the development of this condition.

CKD and HTN are often in a firmly merged relationship as either a cause or a result of one another. Many CKD patients have HTN, and the rate rises when CKD progresses in stages [17]. HTN is responsible for many cardiovascular diseases and high mortality rates in CKD patients, especially those on hemodialysis. In Egypt, hypertensive nephropathy is considered one of the major causes of the prevalence of dialysis as a renal replacement therapy [18].

Many genetic variants increase the risk of kidney disease and kidney failure in non-diabetic, hypertensive adults with African progenitors [19]. Renalase gene polymorphism is one of such genes. One of the renalase variants is the missense polymorphism in the flavin-adenine dinucleotide-binding domain (Glu37Asp) (rs2296545), which displayed an association with HTN [20]. Renalase controls blood pressure by manipulating catecholamine metabolism [21].

In the present study, we assessed Egyptian CKD patients (hypertensive and normotensive) to show the relation between renalase gene (rs2296545) polymorphism and HTN in these patients. Renalase gene (rs2296545) polymorphism is related to the development of CKD and hypertensive CKD. A previous study proved that recombinant renalase has powerful hypotensive effects through cardiac contractility decreasing and slowing heart rate. Renalase supplementation can prevent the development of renal impairment and further organ injury [22].

CC genotypes and C allele carriers had a significant risk of having CKD in comparison to a control group (OR =9.4, 95%CI= 1.2-77.2, P=0.036) and (OR=3.78, 95%CI 1.57-9.08, P=0.003) respectively and also more likely to have hypertensive CKD (OR=13.7, 95%CI 1.5-121.4, P=0.018), (OR=5.04, 95%CI 1.96-12.9, P=0.001). George et al. revealed that renalase polymorphism was associated with CKD and its progression to ESRD in Africans in their systematic review [23].

Malyszko et al. demonstrated the association between renalase and HTN in hemodialysis patients because of an overactive sympathetic nervous system [24]. Lv et al., in their meta-analysis, deduced that renalase (rs2296545) was associated with HTN development risk [25].

In renalase (rs2296545) SNP, at codon 37, glutamic acid is replaced by aspartic acid; this substitution is critical as it affects the affinity of the enzyme for NADH (nicotinamide adenine dinucleotide + hydrogen) [9], which is an essential coenzyme in various biological reactions. The alteration in NADH affinity can impair the enzymatic activity of renalase. Specifically, the reduced affinity for NADH can lead to a disruption in the normal metabolism of catecholamines, which leads to blood pressure elevation and bad outcomes of cardiovascular diseases [9,25].

In our study, we unearthed a striking correlation between the CC genotype of renalase (rs2296545) and blood pressure readings in both CKD patients and those with hypertensive CKD. On the contrary, Shi and Wang concluded that renalase polymorphism was not associated with risk of HTN in all genetic models in their meta-analysis and confirmed that pooling data through meta-analysis increases the statistical power and the strength of their result [7]. Fava et al.'s research proposed that the renalase (rs2296545) variant didn't wield any significant influence over blood pressure levels or the prevalence of HTN [26]. On a similar note, Malyszko et al. echoed this sentiment, concluding that the same genetic variant didn't seem to play a role in determining the values of high blood pressure [20]. Far et al.'s findings suggest a lack of association between the Glu37Asp polymorphism and HTN, perhaps due to the stringent control of blood pressure observed in cases of ischemic heart disease [9].

These conflicting results have sparked much debate among the scientific community, hinting at the possibility of divergent outcomes stemming from the differences in the populations under study. This intriguing discovery underscores the complexity involved in unraveling the genetic underpinnings of blood pressure regulation and the development of HTN. It challenges us to delve deeper into the intricacies of these genetic variants and their interplay with diverse physiological contexts, paving the way for a more nuanced understanding of this intricate puzzle.

There are some limitations to the present study. The small sample size and collection from a single location do not reflect the whole Egyptian population. In addition, using regression analysis in studying the genetic association of renalase gene (rs2296545) polymorphism and HTN was a better statistical choice.

## Conclusions

From the results of this study and due to the statistically significant increase of C allele and CC genotypes in CKD and hypertensive CKD groups more than in the control group, the C allele is considered an independent risk factor for CKD and hypertensive CKD compared to G allele, which is a protective allele. Also, this study concluded that renalase gene polymorphism (rs2296545) was found to be significantly associated with increased risk of CKD and hypertensive CKD in Egyptian patients and can be used to predict the susceptibility of such diseases among Egyptian populations.
